# Construction of Ru(II) Polypyridyl Based Macrocycles: Synthesis, Characterization, Electrochemical, Li^+^ Binding, Antitumour and Anti-HIV properties

**DOI:** 10.1155/MBD.2001.113

**Published:** 2001

**Authors:** Lallan Mishra, Ragini Sinha, P. C. Pandey

**Affiliations:** 1 Department of Chemistry Banaras Hindu University Varanasi 221 005 India

## Abstract

Some ruthenium (II) polypyridyl complexes with a bis-chalcone (obtained by the condensation of 3-methyl-thiophene-2-carboxaldehyde and 4-acetyl pyridine) have been synthesized and characterized spectroscopically (IR, NMR, UV/Vis), conductimetric, elemental analysis and FAB mass data. Their luminescent, redox and Li^+^ binding properties have been studied. The anti-HIV and antitumour activities have also been reported.


### CONSTRUCTION OF Ru(II) POLYPYRIDYL BASED MACROCYCLES: SYNTHESIS, CHARACTERIZATION, ELECTROCHEMICAL, $Li^+$ BINDING, ANTITUMOUR AND ANTI-HIV PROPERTIES

Lallan Mishra*, Ragini Sinha and P. C. Pandey

Department of Chemistry, Banaras Hindu University, Varanasi - 221 005, India

**Abstract**

Some ruthenium (II) polypyridyl complexes with a bis-chalcone (obtained by the condensation of 3-methyl-thiophene-2-carboxaldehyde and 4-acetyl pyridine) have been synthesized and characterized spectroscopically (IR, NMR, UV/Vis), conductimetric, elemental analysis and FAB mass data. Their luminescent, redox and $Li^+$ binding properties have been studied The anti-HIV and antitumour activities have also been reported..

**Introduction**

Studies of ruthenium (II) polypyridyl complexes have been interesting owing to their unique photophysical and redox characteristics [1]. In fact, these properties have led to the development of new ruthenium polypyridyl systems either by incorporating the desired groups within the bipyridine moiety itself or by using other type of donor sites along with the $[Ru(bpy/phen)_2]$ core to form mixed ligand tris-chelates modulating the photo-redox activities of this class of complexes.

Furthermore, it has been reported that Ru(II) complexes bind DNA and a correlation between their binding affinity and activities have already been established [2,3]. Recently a detailed accounts of such properties of ruthenium complexes have been reviewed by Clarke *et al.*[4]. Based on antiviral [5], antifungal [6], antibacterial [7] and antitumour [8] activities of chalcones very recently, several Ru(II) complexes bearing bis-chalcones synthesized by us [9] have been evaluated for their antitumour and anti-HIV activities [10] and some of them have shown quite interesting properties. It was therefore considered worthwhile to explore some new Ru(II) complexes bearing chalcones for the evaluation of their antitumour and anti-HIV properties. The $Li^+$ binding properties of newly synthesized complexes containing 14-membered ring are also studied.

**Materials and Methods**

All the solvents purchased from Merck were distilled using standard procedures prior to use. 3-Methyl-thiophene-2-carboxaldehye, 4-acetyl pyridine, 2,2'-bipyridine and 1,10-phenanthroline purchased from Sigma-Aldrich were used as supplied whereas the *cis*-$[Ru(bpy)_2Cl_2]$ and *cis*-$[Ru(phen)_2Cl_2]$ were prepared by a reported [11] procedure. Tetra-n-butylammonium bromide supplied by Merck was converted into pure tetrabutyl ammonium perchlorate (TBAP) by an available procedure [12]. **Caution:** TBAP could be explosive so the use of small amounts of it is recommended. Neutral alumina for column chromatography was supplied by Merck and used as such. All the reactions were carried out under $N_2$ atmosphere

Microanalysis (C, H and N) performed on a Carlo Ebra Elemental Analyzer 1108 and FAB mass data using a JEOL SX-102 mass spectrometer were carried out at the Central Drug Research Institute, Lucknow, India. IR (KBr Pellets) and UV/Vis data were obtained using a JASCO FT IR 5300 spectrometer and a Shimadzu UV-1601 spectrophotometer respectively, $^1H$ and $^{13}C$ NMR (DMSO-$d_6$) were recorded on a JEOL FX 90Q spectrophotometer at B.H.U., Varanasi, India and $^1H$-$^1H$ COSY (DMSO-$d_6$) spectra was recorded at TIFR, Mumbai, India. Electrochemical measurements were made using an electrochemical interface SI 1287 potentiostat, using a graphite disc as working electrode, a platinium wire auxiliary electrode and $Ag/Ag^+$ reference electrode in a three electrode configuration. All electrochemical experiments were performed after bubbling the solution with dinitrogen for 0.25 h. Solution ($CH_3CN$) emission spectra were measured at University of Bristol, UK. The antitumour and anti-HIV activities were evaluated at the School of Pharmacy, University of North Carolina, USA.

**Synthesis of Ligand (L)**

The ligand was prepared by addition of a solution of 4-acetyl pyridine (0.02 M, 2.2 mL) in ethanol (5 mL) to an ethanolic solution (10 mL) of 3-methyl-thiophene-2-carboxaldehyde (0.02 M, 2.2 mL) containing aqueous NaOH (12 mL, 50%) following reported procedure [5]. After 72 h of stirring distilled water (20 mL) was added to the reaction mixture and then neutralised with dil HCl (10%). The brick-red precipitate formed was filtered and washed several times with $H_2O$ followed by EtOH and was purified by column chromatography on alumina with $CH_3CN$ as eluent. Two dark red and brown fractions were collected. Minor dark red fraction was discarded while the major brown fraction was concentrated by evaporation *in vacuo*. The solid (L) was crystallized from ethanol and dried *in vacuo*, M.P. 90 ± 1°C.

**Synthesis of complexes**

[RuL(bpy)$_2$].(PF$_6$)$_2$, (*1*): An ethanolic solution (10 mL) of *cis*-[Ru (bpy)$_2$Cl$_2$] (bpy = 2,2'-bipyridine) (1 mM, 0.520 g) was mixed to an ethanolic solution (10 mL) of (*L*) (1 mM, 0.350 g) in a 1:1 molar ratio while stirring and the resulting mixture was refluxed for 24 h then cooled to room-temperature and filtered. To the filtrate, a saturated methanolic solution of $NH_4PF_6$ was added. Solid thus obtained was filtered and washed with $H_2O$ and MeOH, and purified on an alumina column with MeCN-saturated aqueous $KNO_3$-water (7:1:0.5 v/v) as eluent [13]. The eluate was then concentrated to dryness and the residue obtained was dissolved in minimum volume of acetone followed by the excess addition of methanolic solution of $NH_4PF_6$. The solid complex (*1*) was recrystallized (by the addition of diethylether to a solution of complex in acetone) as crystalline material, M.P. > 250°C, yield : 40%.

[RuL(phen)Cl$_2$].(PF$_6$)$_2$, (*2*) was also prepared as reported above by refluxing an ethanolic solution of *cis*-[Ru(phen)$_2$Cl$_2$] (where phen = 1,10-phenanthroline) (1 mM, 0.532 g) with an ethanolic solution (10 mL) of (L) (1 mM, 0.350 g).

The elemental analysis and FAB-mass data alongwith other properties of ligand and complexes are shown in Table I.

**Results and Discussion**

The synthesis of ligand has been considered in view of Michael addition [14] (Scheme-I).

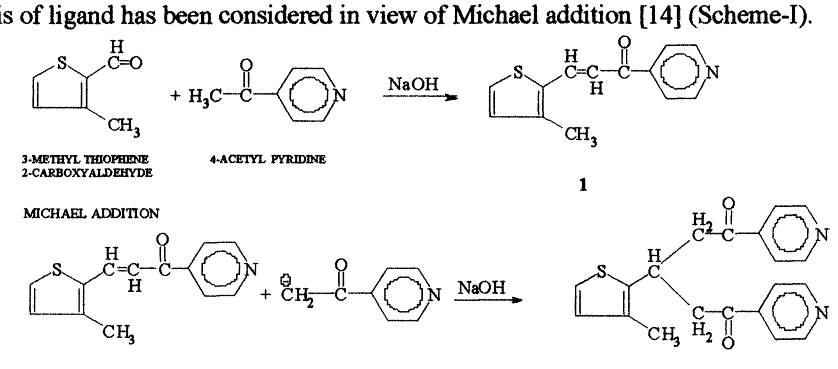

1 **Scheme - I** L

Table I: Physical, analytical and FAB Mass data of ligand and Ru(II) complexes

| Compound | Analysis found (Calc.) | | | m/z | Λ |
|---|---|---|---|---|---|
| | %C | %H | %N | Found (Calcd.) | ($\Omega^{-1}$ cm$^2$mol$^{-1}$) |
| L | 68.03 | 4.63 | 7.95 | 350 (350) | |
| | (68.96) | (4.59) | (8.04) | [M] | |
| [RuL(bpy)$_2$](PF$_6$)$_2$.2Me$_2$CO | 47.74 | 3.95 | 7.55 | 908 (908) | 250 |
| | (47.21) | (3.93) | (7.18) | [M-PF$_6^-$]$^+$ | |
| [RuL(phen)$_2$](PF$_6$)$_2$.2Me$_2$CO | 49.67 | 3.90 | 7.01 | 811 (811) | 280 |
| | (49.30) | (3.77) | (6.9) | [M-2PF$_6^-$]$^{2+}$ | |

The microcrystalline complexes were found thermally stable at room temperature and soluble in acetone, acetonitrile, methanol, ethanol, DMF and DMSO. The molar conductances of the complexes ($CH_3CN$, $10^{-3}$ M) shown in Table 1 is in agreement with the number of counteranions present in the complexes [15]. However, the presence of two moles of acetone in the complex has been considered in view of the fitting of their elemental analysis and observation of a band at ~ 1720 cm$^{-1}$ in their IR spectra.

**IR Spectra**: In the IR Spectra (KBr) of the complexes a band at 1676 cm$^{-1}$ due to the ν(C=O) was found to be similar compared to free ligand (1680 cm$^{-1}$) which indicates that the >C=O group did not participate in the bonding with ruthenium. However, peak observed at 1570 cm$^{-1}$ in the spectrum of the free ligand due to pyridyl moiety shifted to 1545 cm$^{-1}$ and 1500 cm$^{-1}$ in the spectrum of complexes (*1*) and (*2*) respectively, indicating that pyridyl group coordinate to ruthenium.

**$^1$H NMR spectra**: To get further structural support $^1$H NMR spectra of the ligand and its complexes were recorded in DMSO-d$_6$. The $^1$H NMR shows (complex) patterns at δ 9.01 - 8.53, 7.53 - 7.3, 7.06, 6.86, 2.4, 2.00 ppm due to *ortho* and meta-protons of both pyridine rings, HC=CH (thiophene), -C-CH, -CH$_2$ and -CH$_3$ protons respectively. Its $^{13}$C NMR spectrum also showed peaks at δ 185.2 (>C=O), 150.3 (C$_2$ C$_2$'-py), 149.8 (C$_6$, C$_6$'-py), 129.8 (C$_4$, C$_4$'-py), 128.2 (C$_3$, C$_3$'-py), 126.0 (C$_5$-thio), 123.8 (C$_2$-thio), 121.8 (C$_4$-thio), 121.7 (C$_3$-thio), 50.6 (-CH$_2$), 48.9 (-CH), 33.4 (-CH$_3$). The $^1$H NMR spectra of the complexes show resonances at δ 8.75 to 8.6 ppm due to pyridyl protons. Peaks observed in the spectrum of complex (*1*) at δ 8.34 - 8.19, δ

8.09 - 8.02, δ 7.9 - 7.89 and δ 7.61 - 7.42 ppm were assigned to 3,3′, 4,4′, 5,5′ and 6,6′ protons of the bipyridine ring and the bands at δ 9.2, 8.6, 8.37, 8.2 - 7.86 and 7.6 ppm in the spectrum of complex (*2*) were assigned to $H_2/H_9$, $H_4/H_7$, $H_5/H_6$ and $H_3/H_8$ protons of the phenanthroline ring in agreement with an earlier report [15].

The $^1H$ - $^1H$ COSY spectrum of one of a representative complex (*1*) showed that the ortho and meta protons of both pyridine rings couple with the 6,6′-protons of bipyridine. The COSY spectrum also showed weak coupling between $-CH_3$ and $-CH_2$ protons supporting the quartet and triplet observed at δ 2.20 and 2.5 ppm respectively for these protons.

**UV-Vis spectra**: UV-Vis spectra of the complexes in $CH_3CN$ solution ($10^{-3}$ M) were recorded in the range 200 - 800 nm and showed intra-ligand charge-transfer peaks at λ 264, 290 (*1*) and λ 246 nm (*2*) whereas broad peaks observed in the respective spectra at $\lambda_{max}$ 485 and 417 nm were assigned to the $t_2g(Ru) \rightarrow \pi^*$ (ligand) transition.

Thus, on the basis of spectroscopic (IR, $^1H$ NMR, UV/Vis) and conductimetric data alongwith elemental analysis and FAB mass data, the proposed general structure of the complexes is shown in Figure 1.

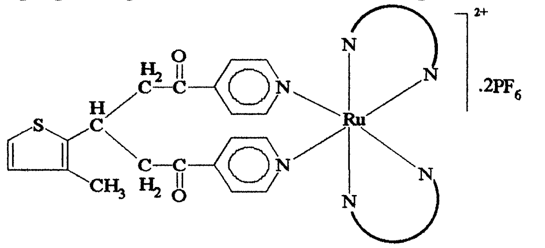

N-N = 2,2′-bipyridine, (*1*) and 1,10-phenanthroline, (*2*)

**Figure 1:** Proposed structures of Ru(II) complexes

**Room temperature emission spectra**

Emission properties of the complexes have been studied in acetonitrile solution ($10^{-6}$ M) at room temperature (excitation at 450 nm). Complexes did not luminesce most likely due to the efficient migration of the electron/energy [16-18] from the triplet excited state of $Ru^*$ to the ligand, as probability of non-radiative emission was discarded since complex (*2*) weakly emitted.

**Redox Properties**: Redox properties of the ruthenium complexes have been studied in acetonitrile solution ($10^{-3}$ M) in the potential range ± 2V using $Ag/Ag^+$ as reference and graphite disc as working electrodes.

**Oxidation**

Redox potential (E°) data are reported in Table II. Cyclic voltammogram of the complex (*1*) showed two oxidation peaks at 0.88 V and 1.45 V, the former peak being considered to arise from a Ru(II) → Ru(III) oxidation as the latter peak lies in the potential range of ligand oxidation in view of the fact that upon coordination peaks could be shifted towards less positive potentials. Similarly, in the cyclic voltammogram (CV) of complex (*2*) these oxidations occurred at 0.92 V and 1.43 V.

**Reduction**

During the reduction of the free ligand two peaks were observed at -0.85 and -1.2 V. However, four reduction peaks were observed at -0.8, -1.1, -1.35 and -1.55 V in the cyclic voltammogram of complex (*1*). The reduction peaks at -0.8 and -1.1 V were assigned to arise from ligand reduction whereas the peaks at -1.35 and -1.55 V could be considered to arise from bipyridine reduction in view of earlier report [19]. Similarly four reduction peaks at -0.45, -1.2, -1.35 and -1.8 V also arise upon reduction of the complex (*2*) which were in consistence with the earlier report [20].

**$Li^+$ binding studies**: Due to the presence of a 14-membered ring cavity in the complexes, their binding with $Li^+$ on a preliminary level was studied using UV/Vis and electrochemical techniques.

The presence of LiCl ($10^{-4}$ M, $CH_3CN$) in the solution of the complexes in acetonitrile ($10^{-5}$ M) shifted both the MLCT and intra-ligand transitions towards higher energies along with the increase in their absorbances (Table III).

Table II: Electrochemical data* of ligand and Ru(II) complexes in MeCN at 298 K

| | Redox potential $E^\circ$ 298 / V vs Ag/AgCl | | |
|---|---|---|---|
| | Oxidations | | Reductions |
| | $Ru^{3+}$ / $Ru^{2+}$ | Ligand | |
| Ligand | | 1.55 | -0.85, -1.2 |
| $[Ru.L(bpy)_2]^{2+}$ | 0.88 | 1.45 | -0.4, -1.15, -1.45, -1.75 |
| $[Ru.L(phen)_2]^{2+}$ | 0.92 | 1.40 | -0.45, -1.2, -1.35, -1.8 |
| $[Ru.L(bpy)_2]^{2+}$ + $10^{-4}$ mol $dm^{-3}$ LiCl | 1.15 | 1.72 | -0.5, -1.08, -1.65 |
| $[Ru.L(phen)_2]^{2+}$ + $10^{-4}$ mol $dm^{-3}$ LiCl | 0.95 | 1.43 | -0.5, -1.45, -1.8 |

* Conditions; solvent, MeCN, supporting electrolyte TBAP (0.1 M); working electrode, graphite disc; auxiliary electrode, platinum; reference electrode Ag/$Ag^+$. Solute concentration ~$10^{-3}$ *M*, $E_{1/2}$ = 0.5 ($E_{pa}$ + $E_{pc}$) where $E_{pa}$ and $E_{pc}$ = anodic and cathodic peak.

Table III: UV-Vis data in MeCN ($10^{-3}$ *M*) at 298 K for Ru(II) complexes in presence and absence of LiCl

| | | $\lambda_{max}$, nm ($10^4$, ε, d $m^3$ $mol^{-1}$ $cm^{-1}$) | |
|---|---|---|---|
| Complex | Transitions | | +LiCl (5 ×$10^{-4}$ mol d $m^{-3}$) |
| $[RuL(bpy)_2]^{2+}$ | π - π* (2)[a] | 246 (7.75) | 242 (8.81) |
| | π - π* (1)[a] | 290 (6.65) | 286 (7.06) |
| | m.l.c.t. | 485 (0.83) | 476 (0.97) |
| $[RuL(phen)_2]^{2+}$ | π - π* (2)[a] | 230 (10.16) | 226 (13.95) |
| | π - π* (1)[a] | 264 (10.69) | 263 (12.04) |
| | m.l.c.t. | 417 (0.75) | 349 (2.98) |
| | | | 413 (1.30) |

[a] (1) and (2) used to denote different ligand orbitals.

**Electrochemical studies of the complexes (*1* and *2*) in the presence of $Li^+$**: As shown in Table III the addition of a $CH_3CN$ solution of LiCl ($10^{-4}$ M) to the complexes ($10^{-3}$ M, $CH_3CN$) shifted metal-based oxidation peak towards a more positive potential by 230 mV in complex (*1*) whereas in complex (*2*) shift was 40 mV compared to LiCl unbound complexes. The larger shift observed for complex (*1*) as compared to complex (*2*) may be considered in view of more flexibility of the bipyridine ring.

Thus, in view of these observations and earlier reports [21] binding of $Li^+$ inside the cavity of the macrocycle could be considered on a preliminary level.

**Antitumour and anti-HIV activities**

The antitumour and anti-HIV activities were evaluated using standard procedure [22,23]. The cytotoxicity data of free ligand alongwith their ruthenium complexes are shown in Table IV. The free ligand showed potential activity against IA9 and A549 which could be considered [24] in view of the significant bioactivities shown by an earlier ligand system bearing similar functional (-N-C=O) group. However, the complexes showed a better activity as compared to the free ligand against almost all tumour cells. This inhibitory behaviour of the complexes may be considered in view of their interaction with DNA as reported [25,26] earlier. Complex (*2*) however showed better activity against IA9, KB-VIN and A-549 as compared to complex (*1*).

From the anti-HIV data of the ligand and complexes as shown in table IV, it could be seen that complex (*1*) show a smaller $IC_{50}$ value than the free ligand. However the $EC_{50}$ value of the free ligand (13.3 μM) showed that ligand is more active than the complex (*1*) which may be understood in terms of steric factors [27]. Complex (*2*) bearing a bulkier group (1,10-phenanthroline) did not show activities (NA = inhibition < or = 5% at 20 g/mL). The activity of these compounds has been compared with the standard AZT (azido-thymidine) treated as control under the similar experimental conditions.

Table IV: Antitumour* and anti-HIV activity of free ligand and its Ru(II) complexes

| Compound | Tumour cells | | | | | | | | Anti-HIV |
|---|---|---|---|---|---|---|---|---|---|
| | HCT-8° | IA9 | HOS | KB | KB-VIN | MCF-7 | A549 | U87-MG | $IC_{50}$ (μM) |
| L | 41.4 | 37.1 | >20 (114) | 46.2 | 50 | 50 | 29.1 | >20 (60) | 48.0 |
| (*1*) | >20(33.3) | 10.3 | >20(23.9) | 14.5 | >20(13.6) | 16.6 | 14.9 | >20 (39.3) | 17.7 |
| (*2*) | 13.9 | 9.7 | 12.3 | 13.9 | 7.2 | 14.8 | 9.0 | >20 (36.1) | - |
| AZT** | - | - | - | - | - | - | - | - | 1870 |

* Values are $IC_{50}$ concentration in μM.
Note, if inhibition < 50% at 20 microgram per mL, percent inhibition observed is the value in brackets.
Tumour/tissue type; HCT - Ileocecal, IA9 - Ovarian, HOS - Bone, KB - Nasopharynx, MCF - Breast, A549 - lung, U87-MG - Glioblastoma.

o = ED - 50 in microgram per mL is less than 50% at highest test concentration.

** AZT = Azido-thymidine; m/z = 267.25

**Acknowledgement**
One of the authors (LM) gratefully acknowledges the receipt of financial assistance from U.G.C., New Delhi, India. Authors are also grateful to Prof. H. Itokawa, University of North Carolina, U.S.A. for evaluating the cytotoxicity and anti-HIV activities of the compounds and Prof. M.D. Ward, University of Bristol, U.K. for the luminescent measurement as well as valuable suggestions in the preparation of the manuscript.

**References**

1. V. Balzani, A. Juris, M. Venturi, S. Campagna and S. Serroni, *Chem. Rev.* 1996, **96**, 759 and references therein.
2. A. Ambroise and B.G. Maiya, *Inorg. Chem.*, 2000, **39**, 4264.
3. K.E. Erkkila, D.T. Odom and J.K. Barton, *Chem. Rev.*, 1999, **99**, 2777.
4. M.J. Clarke, F. Zhu and D.R. Frasca, *Chem. Rev.*, 1999, **99**, 2511.
5. N.D. Meyer, A Haemers, L. Mishra, H.K.Pandey, L.A.C. Pieters, D.A.V. Berghe and A.J. Vlietinck, *J. Med. Chem.*, 1991, **34**, 736.
6. A.K. Bhat, R.P. Bhamaria, M.R. Patil, R.A. Bellare and C.V. Deliwala, *Indian J. Chem.*, 1972, **10**, 694.
7. V.K. Ahluwalia, R.R. Mann, S. Bala, *Indian J. Chem.*, 1989, **28B**, 608.
8. V.K. Ahluwalia, L. Nayal, N. Kaila, S. Bala and A.K. Tehin, *Indian J. Chem.*, 1987, **26B**, 384.
9. L. Mishra and R. Sinha, *Indian. J. Chem.*, 2000, **39A**, 1295.
10. L. Mishra, R. Sinha, H. Itokawa, K.F. Bastow, Y. Tachibana, Y. Nakanishi, N. Kilgore and K.H. Lee, *J. Bioorg. Med. Chem.*, 2001 (Accepted).
11. B.P. Sullivan, D.J. Salmon and T.J. Meyer, *Inorg. Chem.*, 1978, **17**, 3334.
12. D.T. Sawyer, A. Sobkowiak and J.L. Roberts Jr, *Electrochemistry for Chemists*, 2nd Ed., Wiley, New York, 1995.
13. E.C. Constable and A.M.W.C. Thompson, *J. Chem Soc. Dalton Trans.*, 1992, 3467, and references therein.
14. J. March, *Adv. Org. Chemistry, Reactions, Mechanisms and structure*, 4th Ed. John Wiley, Sons, 1992.
15. L. Mishra, A. K. Yadaw, S. Srivastava and A.B. Patel, *New J. Chem.*, 2000, **24**, 505.
16. M.A. Hayes, C. Meckel, E. Schatz and M.D. Ward, *J. Chem. Soc. Dalton Trans.*, 1992, 703;
17. S. Arounagiri and B.G. Maiya, *Inorg. Chem.*, 1999, **38**, 842;
18. L. Mishra, C.S. Choi and K. Araki, *Chem. Lett.*, 1997, 447.
19. L. Mishra, A.K. Yadaw, C.S. Choi and K. Araki, *Indian J. Chem.*, 1999, **38A**, 439 and references therein.
20. A. Lawrence, *Polyhedron*, 1985, **4**, 817.
21. P.D. Beer, *Chem. Soc. Rev.*, 1989, **18**, 409.
22. L.V. Rubinstein, R.H. Shoemaker, K.D. Paull, R.M. Simon, S. Tosini, P. Skehon, D.A. Scudiero, A. Monks, and M.R. Boyd, *J. Natl. Cancer Inst.*, 1990, **82**, 1113.
23. I.C. Sun, J.K. Shen, H.K. Wang, L.M. Cosentino and K.H. Lee, *Bioorg. Med. Chem. Lett.*, 1998, **8**, 1267.
24. R. Garg, S.P. Gupta, H. Gao, M.S. Babu, A.K. Debnath and C. Hansch, *Chem. Rev.*, 1999, **99**, 3525.
25. A.D. Kelman, M.J. Clarke, S.D. Edmonds and H.J. Peresie, *J. Clin. Hematol. Oncol.* 1977, **7**, 274.
26. L. Mishra and A.K. Yadaw, *Indian J. Chem.*, 2000, **39A**, 660.
27. S.P. Gupta, *Chem. Rev.*, 1987, 1184.

**Received: April 17, 2001 – Accepted: May 8, 2001 –**
**Accepted in publishable format: May 15, 2001**

